# Reconciling links between diversity and population stability across global plant communities

**DOI:** 10.1111/nph.70921

**Published:** 2026-01-16

**Authors:** Xiaobin Pan, Yann Hautier, Jan Lepš, Shaopeng Wang, Kathryn E. Barry, Manuele Bazzichetto, Stefano Chelli, Jiří Doležal, Nico Eisenhauer, Franz Essl, Felícia M. Fischer, Oscar Godoy, Daniel Gómez‐García, Lars Götzenberger, Clara Gracia, Anaclara Guido, Lauren M. Hallett, Susan Harrison, Miao He, Andrew Hector, Pubin Hong, Forest Isbell, George A. Kowalchuk, Victor Lecegui, Xiaofei Li, Maowei Liang, Frédérique Louault, Maria Májeková, Rob Marrs, Neha Mohanbabu, Akira S. Mori, Robin J. Pakeman, Alain Paquette, Begoña Peco, Josep Peñuelas, Valério D. Pillar, Marta Rueda, Wolfgang Schmidt, Jules Segrestin, Marta Gaia Sperandii, Enrique Valencia, Vigdis Vandvik, Shengnan Wang, David Ward, Susan Wiser, Ben A. Woodcock, Chong Xu, Truman Young, Fei‐Hai Yu, Liting Zheng, Zhiwei Zhong, Francesco de Bello

**Affiliations:** ^1^ Ecology and Biodiversity Group Utrecht University Padualaan 8 Utrecht 3584 CH the Netherlands; ^2^ Department of Botany, Faculty of Science University of South Bohemia Na Zlaté stoce 1 České Budějovice 370 05 Czech Republic; ^3^ Biology Center of the Czech Academy of Sciences Institute of Entomology České Budějovice CZ‐37005 Czech Republic; ^4^ Institute of Ecology, College of Urban and Environmental Science, and State Key Laboratory of Vegetation Structure, Function and Construction (VegLab) Peking University Beijing 100871 China; ^5^ Department of Biological, Geological and Environmental Sciences University of Bologna Bologna 40126 Italy; ^6^ School of Biosciences & Veterinary Medicine University of Camerino Camerino 62032 Italy; ^7^ Institute of Botany of the Czech Academy of Sciences Dukelská 135 Třeboň 379 01 Czech Republic; ^8^ German Centre for Integrative Biodiversity Research (iDiv) Halle‐Jena‐Leipzig Puschstrasse 4 Leipzig 04103 Germany; ^9^ Institute of Biology Leipzig University Puschstrasse 4 04103 Leipzig Germany; ^10^ Division of BioInvasions, Global Change and Macroecology, Department of Botany and Biodiversity Sciences University of Vienna Rennweg 14 Vienna 1030 Austria; ^11^ Department of Ecology Universidade Federal do Rio Grande do Sul Porto Alegre RS 91501‐970 Brazil; ^12^ Estación Biológica de Doñana (EBD‐CSIC) Americo Vespucio 26 Sevilla 41092 Spain; ^13^ Departamento de Conservación de Ecosistemas Naturales Instituto Pirenaico de Ecología IPE‐CSIC Postbox 64 22700 Jaca Spain; ^14^ Centro de Investigaciones sobre Desertificación (CSIC‐UV‐GV) Carretera CV‐315, Km 10,7 (Ant. Carretera Moncada‐Náquera, km 4.5) Moncada 46113 València Spain; ^15^ Faculty of Biology University of València Burjassot 46100 València Spain; ^16^ Instituto de Ecología y Ciencias Ambientales, Facultad de Ciencias Universidad de la República Montevideo 11400 Uruguay; ^17^ Institute of Arctic and Alpine Research, University of Colorado Boulder CO 80303 USA; ^18^ Department of Biology and Environmental Studies Program University of Oregon Eugene OR 97403 USA; ^19^ Department of Environmental Science and Policy University of California Davis CA 95616 USA; ^20^ Department of Ecology, Evolution, and Behavior University of Minnesota St Paul MN 55108 USA; ^21^ Department of Biology University of Oxford Oxford OX1 3RB UK; ^22^ College of Resources and Environmental Sciences Jilin Agricultural University Changchun 130118 China; ^23^ Cedar Creek Ecosystem Science Reserve University of Minnesota 2660 Fawn Lake Dr NE East Bethel MN 55005 USA; ^24^ Université Clermont Auvergne INRAE, VetAgro Sup, UREP Clermont‐Ferrand 63000 France; ^25^ Plant Ecology Group University of Tübingen Auf der Morgenstelle 5 Tübingen 72076 Germany; ^26^ School of Environmental Sciences University of Liverpool Liverpool Merseyside L69 3GP UK; ^27^ Copernicus Institute of Sustainable Development, Utrecht University Princetonlaan 8A Utrecht 3584 CB the Netherlands; ^28^ Research Center for Advanced Science and Technology The University of Tokyo 4‐6‐1, Komaba, Meguro Tokyo 153‐8904 Japan; ^29^ The James Hutton Institute Craigiebuckler Aberdeen AB15 8QH UK; ^30^ Centre for Forest Research Université du Québec à Montréal C.P. 8888, Succursale Centre‐ville Montréal QC H3C 3P8 Canada; ^31^ Terrestrial Ecology Group (TEG), Department of Ecology Institute for Biodiversity and Global Change, Universidad Autónoma de Madrid 28049 Madrid Spain; ^32^ CREAF Bellaterra (Cerdanyola del Vallès) Catalonia 08913 Spain; ^33^ CSIC Global Ecology Unit, CREAF‐CSIC‐UAB Bellaterra Barcelona Catalonia 08193 Spain; ^34^ Department of Plant Biology and Ecology Universidad de Sevilla C/Profesor García González s/n Sevilla 41012 Spain; ^35^ Department of Forest Silviculture and Forest Ecology University of Göttingen Göttingen DE‐37077 Germany; ^36^ Department of Science University of Roma Tre Rome 00146 Italy; ^37^ Departamento de Biodiversidad, Ecología y Evolución, Facultad de Ciencias Biológicas Universidad Complutense de Madrid Madrid 28040 Spain; ^38^ Department of Biological Sciences University of Bergen Thormøhlensgate 53 Bergen 5006 Norway; ^39^ Bjerknes Centre for Climate Research, University of Bergen Allégaten 70 Bergen 5007 Norway; ^40^ Department of Biological Sciences Kent State University Kent OH 44242 USA; ^41^ Manaaki Whenua – Landcare Research 74 Gerald St Lincoln 7608 New Zealand; ^42^ UK Centre for Ecology and Hydrology Maclean Building, Crowmarsh Gifford Wallingford OX10 8BB UK; ^43^ State Key Laboratory of Efficient Utilization of Arable Land in China Institute of Agricultural Resources and Regional Planning, Chinese Academy of Agricultural Sciences Beijing 100081 China; ^44^ Key Laboratory of Arable Land Quality Monitoring and Evaluation, Ministry of Agriculture and Rural Affairs Institute of Agricultural Resources and Regional Planning, Chinese Academy of Agricultural Sciences Beijing 100081 China; ^45^ Department of Plant Sciences University of California Davis Davis CA 95616 USA; ^46^ School of Life and Environmental Sciences Shaoxing University Shaoxing 312000 Zhejiang China; ^47^ Institute of Wetland Ecology & Clone Ecology Taizhou University Taizhou 318000 Zhejiang China; ^48^ Institute of Grassland Science, Key Laboratory of Vegetation Ecology of the Ministry of Education, Songnen Grassland Ecosystem National Observation and Research Station Northeast Normal University Changchun 130024 China; ^49^ Key Laboratory of Grassland Resources (Inner Mongolia Agricultural University), Ministry of Education Hohhot 010021 China

**Keywords:** average richness, biodiversity, cumulative richness, dominance, populations, rare species, unweighted and weighted population stability

## Abstract

- Maintaining ecological stability is essential for sustaining ecosystem functions and the benefits they provide to society. Ecological theory predicts that plant diversity destabilizes local populations, yet empirical studies report variable effects.
- We hypothesize that this discrepancy arises at least in part from differences captured by different diversity (average vs cumulative richness, i.e. the mean annual richness vs the cumulative richness across years) and stability metrics (abundance‐unweighted vs weighted mean population stability). To test this, we analyzed data from > 8000 permanent vegetation plots across biomes on five continents.
- We found a negative (i.e. destabilizing) diversity–stability relationship when using abundance‐weighted rather than unweighted measures of population stability, which are more influenced by dominant species. Similarly, cumulative richness – capturing total species occurrence over time and long‐term turnover – reveals a stronger destabilizing effect compared to average annual richness.
- Our findings reveal that, when specific metrics of diversity and stability are considered, more species and potentially the associated increase in interspecific competition tend to destabilize populations across natural ecosystems world‐wide – particularly those of dominant species.

## Introduction

The diversity–stability relationship has been the subject of longstanding debate, and understanding its drivers remains a central focus in ecology (MacArthur, 1955; Goodman, 1975; Loreau & de Mazancourt, 2013; de Bello *et al*., 2021). Early ecological theory suggested that complex, diverse ecosystems are inherently more stable than simpler ones (Odum & Eugene, 1953; MacArthur, 1955; Elton & Charles, 1958). Later theoretical work, however, challenged this idea, demonstrating that diversity could have a destabilizing effect (Gardner & Ashby, 1970; May, 1972). Subsequent studies of plant productivity reconciled these views by showing that the diversity–stability relationship depends on the level of organization: plant diversity enhances the temporal invariability of productivity at the community level (i.e. community stability) but reduces it at the population levels (i.e. population stability) (Tilman, 1996; Caldeira *et al*., 2005; Tilman *et al*., 2006; Hector *et al*., 2010). It is now widely accepted that species diversity increases community stability (Cardinale *et al*., 2012; Hautier *et al*., 2015; Isbell *et al*., 2015; Xu *et al*., 2021; Liang *et al*., 2022), though these effects may be weaker in natural vs experimental settings (Blüthgen *et al*., 2016; van der Plas, 2019; Valencia *et al*., 2020; Lisner *et al*., 2024). However, considerable debate remains over whether species diversity increases or decreases population stability. In particular, a recent meta‐analysis reported that population stability increases with diversity in observational studies but decreases with diversity in experimental studies (Xu *et al*., 2021).

Such inconsistency in diversity–population stability relationships may reflect the distinct ecological processes captured by the metrics used to measure both plant diversity and population stability. For example, the methodology used to estimate population stability differs between studies. Since Tilman's (1996) seminal work, population stability has commonly been assessed as the inverse of the coefficient of variation in the abundance of individual species within a community (McGrady‐Steed & Morin, 2000; Romanuk *et al*., 2006; Tilman *et al*., 2006). To account for variations across individual species (Romanuk & Kolasa, 2004), population stability was later calculated by averaging the stability across species within a community, that is, unweighted mean population stability (Steiner *et al*., 2005; Romanuk *et al*., 2009; Houlahan *et al*., 2018). Campbell *et al*. (2011) found that species richness has a stabilizing effect when population stability is calculated as the mean stability across all populations, whereas assessment within individual populations is more likely to yield a destabilizing effect. Further theoretical developments have suggested that community stability is more closely related to the weighted mean population stability, as the stability of dominant species tends to drive overall community patterns (Thibaut & Connolly, 2013; Wang & Loreau, 2014, 2016).

**Fig. 1:**
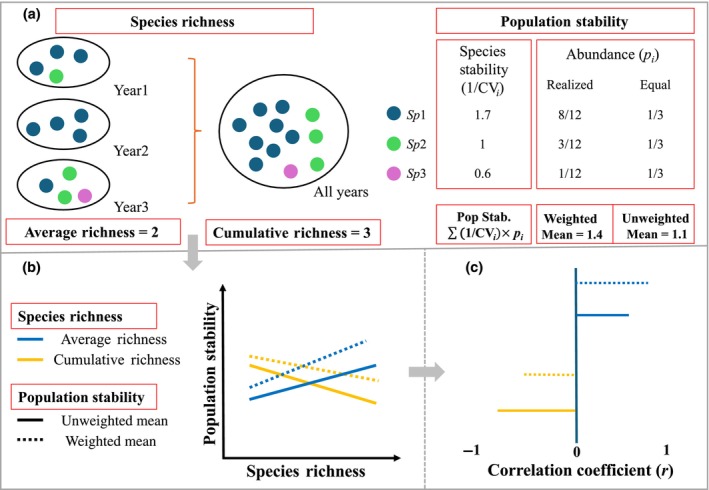
Conceptual diagram illustrating (a) the concept of species diversity (average richness, mean annual richness; cumulative richness, cumulative richness across years), and population stability metrics (unweighted mean, weighting species by taking them equally; weighted mean, weighting species by their realized mean relative abundance across all years); (b) relationships between plant species diversity and population stability, considering the emergent properties of species diversity and population stability metrics; and (c) the expected correlation coefficients (*r*) between plant diversity and population stability based on the relationships in (b). Correlation coefficients are used as they are not affected by sampling size, allowing for comparisons between datasets.

The key difference between the unweighted and weighted mean population stability is that the latter assigns proportionally greater weight to dominant species (Fig. 1a). Empirical studies have often found that dominant species tend to be more stable than non‐dominant ones – an effect not merely due to sampling artifacts but reflecting biological mechanisms, for instance the buffering effect of large populations against demographic stochasticity (Bai *et al*., 2004; Roscher *et al*., 2011; Wang *et al*., 2020). This pattern aligns with Taylor's power law (Taylor, 1961), which describes a scaling relationship between the variance and the mean of population abundance across time or space. According to this law, species with higher mean abundance tend to have proportionally lower variability when the scaling factor is < 2, resulting in greater temporal stability (Segrestin *et al*., 2024). If this is the case, as species diversity increases, the greater inclusion of non‐dominant, but unstable species would result in a more negative diversity–population stability relationship when using unweighted population stability (compare solid and dashed lines of the same color in Fig. 1b,c).

How species diversity is estimated also differs across studies. Some studies use ‘cumulative richness’ – the total number of species recorded in a plot across all sampling years within a time series (Tilman *et al*., 2006; Hector *et al*., 2010; Roscher *et al*., 2011). Others use ‘average richness’ – defined as the mean number of species observed at a location, calculated by averaging annual species richness across years (Houlahan *et al*., 2018; Liang *et al*., 2025). It is important to stress that these measures likely emphasize slightly different ecological processes (Hagan *et al*., 2021; Allan *et al*., 2025). Cumulative richness includes rare or transient species that are not consistently present in a community but better reflect the potential pool of species that coexist across years. As a result, some included species likely exhibit strong fluctuating abundance over time. Higher cumulative richness may therefore amplify population fluctuations (Tilman, 1999; Lehman & Tilman, 2000), resulting in a negative cumulative richness–population stability relationship (orange lines in Fig. 1b,c). By contrast, average richness represents the typical number of species co‐occurring at a given time. It captures the result of community assembly due to local biotic interactions (e.g. competition and facilitation) and environmental filtering under particular annual conditions. It is also important to stress that the relationship between diversity and population stability can differ partly due to mathematical constraints linked to the indices considered. For example, the ratio of cumulative to average richness could represent a sort of temporal turnover. It should increase with more species absences over time. Additionally, species with frequent absences are typically those with lower abundance and stability (Supporting Information Notes S1). As a result, higher temporal compositional turnover can be associated with lower population stability. This pattern is likely stronger for unweighted population stability, which is more influenced by rare species. Therefore, the relationship between population stability and cumulative richness is expected to be more negative, or at least less positive, than with average richness (blue lines in Fig. 1b,c).

Beyond differences in diversity and stability metrics, the strength and direction of the diversity–population stability relationship can vary considerably among ecosystems, experiments, and study designs. For instance, climatic conditions and disturbance regimes can alter species interactions and the relative importance of dominant vs rare species (Loreau & de Mazancourt, 2013). Likewise, experimental design, such as the type of abundance metric (biomass, cover, or frequency), plot size, and study duration, may influence how temporal stability is captured (Campbell *et al*., 2011). Understanding how these abiotic and biotic factors jointly modulate the relationship is therefore essential to reconcile conflicting evidence and identify general ecological drivers.

In this context, we examined the empirical relationships between plant species diversity and population stability across natural and semi‐natural systems spanning diverse vegetation types on five continents. Specifically, we compared two measures of species diversity: (1) average richness (the average annual species richness across years) and (2) cumulative richness (the total number of species recorded in a plot across all years), alongside two measures of population stability (abundance‐unweighted vs abundance‐weighted mean population stability). We expect (Fig. 1) that inconsistencies across results can be attributed at least in part to different indices considered in measuring species richness and mean population stability, with more destabilizing effects when considering the role of non‐dominant species (unweighted population stability) and the total amount of species found in a location (cumulative richness) compared to weighted population stability and annual richness. We further tested the degree to which the diversity–population stability relationship varies across abiotic and biotic gradients.

## Materials and Methods

### Datasets

We used 88 datasets from the LOTVS collection of temporal vegetation data (https://lotvs.csic.es). This comprehensive collection comprises data for > 11 000 permanent plots of natural and semi‐natural vegetation that have been consistently sampled for periods ranging between 6 and 99 yr. None of the plots included in our analysis was part of a manipulated biodiversity experiment. After excluding plots with multiple sampling events within the same year, we retained 8243 plots for analysis. These data were collected from study sites across various vegetation types (forest understory, grassland, and shrubland) on five continents (Europe, America, Africa, Asia, and Australia) (Fig. S1). These datasets vary in their sampling methodology (e.g. aboveground biomass, visual species cover, and species frequency), plot size, duration of sampling, and management regimes (e.g. grazing, fertilization, and burning), which allows for more generalizable conclusions. Climatic conditions also vary widely, with mean annual precipitation ranging from 140 to 2592 mm and mean annual temperatures from −11.5 to 20.1°C. More details on LOTVS are available in Sperandii *et al*. (2022).

### Estimation of population stability and richness

We quantified temporal stability as the invariability of population abundance (Tilman *et al*., 2006). Specifically, we computed the coefficient of variation (CV), defined as the SD of population abundance divided by its temporal mean across years. To account for directional changes in species abundance over time, we also calculated detrended population CV using the three‐term local variance method (Lepš *et al*., 2019). Within each plot, species that occurred in fewer than 30% of sampling years (e.g. species that appeared for fewer than 3 yr in plots sampled over a 10‐yr period) were excluded from the following analysis, since measures of temporal stability can be extremely volatile with data with excessive zeros (Májeková *et al*., 2014; Valencia *et al*., 2020). We also applied 10% and 20% species exclusion thresholds to demonstrate that our results are robust to the choice of threshold. We calculated abundance‐unweighted and weighted mean population CV (hereafter UPCV and WPCV, respectively) for each plot as:

$$\text{UPCV}=\frac{1}{S}\sum_{i=1}^{S}\frac{\sigma_{i}}{\mu_{i}}$$

$$\text{WPCV}=\sum_{i=1}^{S}\frac{\sigma_{i}}{\mu_{i}}\times p_{i}$$

where *S* is the number of species in the plot, $\mu_{i}$ is the mean abundance of species *i* across years, $\sigma_{i}$ is the SD of population abundance for species *i*, and $p_{i}$ is the relative abundance of species *i*.

To assess the influence of relative abundance on the diversity–population stability relationship, we classified species based on their mean abundance across all sampling years: dominant species as the most abundant one within a community, rare species as those with a mean relative abundance of < 5%, and subordinate species as all others (Mouillot *et al*., 2013; Avolio *et al*., 2019). We then calculated unweighted mean population CV for dominant, subordinate, and rare species, respectively (hereafter UPCV_D, UPCV_S, and UPCV_R).

To quantify species richness over time, we calculated both average and cumulative richness for each plot. Average richness refers to the mean number of species observed in a plot across all sampling years, while cumulative richness is the total number of unique species recorded in a plot over the same period. Temporal turnover was then defined using the classical multiplicative approach as the ratio of cumulative to average richness (Whittaker, 1972).

### Data analysis

We first performed Pearson correlation tests for each dataset to estimate relationships between species diversity (average and cumulative richness) and population stability (UPCV and WPCV) across plots. Correlation tests were used because the correlation coefficient (*r*) is unaffected by sample size, enabling meaningful comparisons between datasets. All parameters were log‐transformed to improve normality. To ensure reliability, we excluded datasets with fewer than five plots, leaving 84 datasets. One additional dataset was removed for cumulative richness because it showed no variation across plots. For each combination of diversity and population stability metrics, we calculated the correlation coefficient (*r*) for each dataset. To ensure consistency in interpretation, we reversed the sign of *r* values so that positive r values always indicate a positive relationship between diversity and stability (Xu *et al*., 2021).

To analyze whether the diversity–population stability relationship depends on the metrics used, we utilized (1) linear mixed‐effects models with *r* values as response variable, metrics of species diversity, of population stability, and their interaction as explanatory variables, and dataset as a random factor, and (2) random‐effects meta‐regression models fitted with restricted maximum likelihood for each metric combination. For the meta‐regression models, effect sizes were Fisher's *Z*‐transformed correlation coefficients, with sampling variances calculated based on the number of plots per dataset. As both approaches produced similar results, we report only the outcomes from the linear mixed‐effects models in the main text. We applied the same approach to examine the relationships between species diversity (average and cumulative richness) and the temporal stability of dominant, subordinate, and rare species (UPCV_D, UPCV_S, and UPCV_R), and extracted the corresponding *r* values. We then tested whether these *r* values were associated with those from the correlations between species diversity and overall population stability (UPCV and WPCV). We also tested the correlation between temporal turnover and population stability (UPCV and WPCV). To further explore dynamics among different groups, we calculated the mean population size (i.e. mean abundance across years) for dominant, subordinate, and rare species in each plot and assessed how their mean population size responded to increasing diversity.

Finally, we evaluated how the strength of the diversity–population stability relationship varied among the datasets. We ran linear models for each combination of diversity and population stability metrics to test the effects of abiotic and biotic variables on *r* values. The explanatory variables included type of abundance metric (biomass, frequency, or cover), plot size (m^2^), study duration (sampling years), management coverage (percentage of control plot within the dataset), mean average richness, and two climate axes derived from principal component analysis (data from Gracia *et al*., 2026, 71.6% of the total variance was explained by the first two axes): climate PC1 (dryness and coldness) and climate PC2 (extreme climate events). We used cover as the reference level for the abundance metric, and log‐transformed plot size and study duration to correct for skewness. Variance inflation factors for all abiotic and biotic predictors were below 2, indicating no problematic multicollinearity among these variables.

The analyses and graphical outputs were performed using R software v.4.4.1(R Core Team, 2024), with the following packages: emmeans (Lenth, 2024), ggplot2 (Wickham, 2016), lme4 (Bates *et al*., 2015), metafor (Viechtbauer, 2010), patchwork (Pedersen, 2024), tidyverse (Wickham *et al*., 2019), and vegan (Oksanen *et al*., 2024).

## Results

**Fig. 2:**
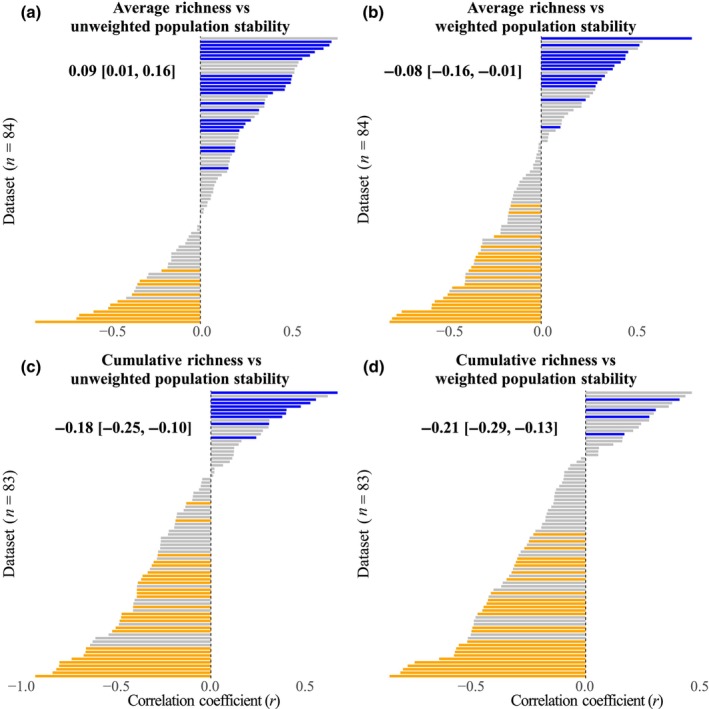
Histograms of the correlation coefficients (*r*) for each dataset between average richness and unweighted population stability (a), average richness and weighted population stability (b), cumulative richness and unweighted population stability (c), and cumulative richness and weighted population stability (d). Different numbers of datasets were used due to the selection process. Significant positive, significant negative, and nonsignificant correlations are represented by blue, orange, and gray colors, respectively. Mean correlation coefficients ($\bar{r}$) and 95% confidence intervals from linear mixed‐effects models are also shown within each panel.

The relationship between plant diversity and population stability strongly depended on the choice of diversity and population stability metrics. A linear mixed‐effects model confirmed this, identifying an interaction between diversity and population stability metrics on the correlation coefficient (*r*) (*F*_1,246.4_ = 12.73, *P* < 0.001; Table S1). The relationship between average richness and unweighted population stability was positive ($\bar{r}$ = 0.09, *P* = 0.026). Fifty‐three of the 84 datasets showed a positive relationship, with 21 being significant (Fig. 2a). By contrast, the relationship between average richness and weighted population stability was, on average, negative ($\bar{r}$ = −0.08, *P* = 0.030; Fig. 2b). We observed predominantly negative relationships between cumulative richness and population stability (Fig. 2c,d), with a weaker relationship for unweighted ($\bar{r}$ = −0.18, *P* < 0.001) than for weighted population stability ($\bar{r}$ = −0.21, *P* < 0.001). Results remained consistent when using detrended population CV (Table S2) and when applying 10 and 20% exclusion thresholds (Tables S3, S4). Within datasets, changes in *r* values from average to cumulative richness were consistently negative for unweighted population stability (Fig. S2A) and mostly negative for weighted population stability (Fig. S2B), meaning that within‐site relationships became consistently more negative when using cumulative richness. However, the change from unweighted to weighted population stability was generally negative for average richness (Fig. S2C) but not for cumulative richness (Fig. S2D).

**Fig. 3:**
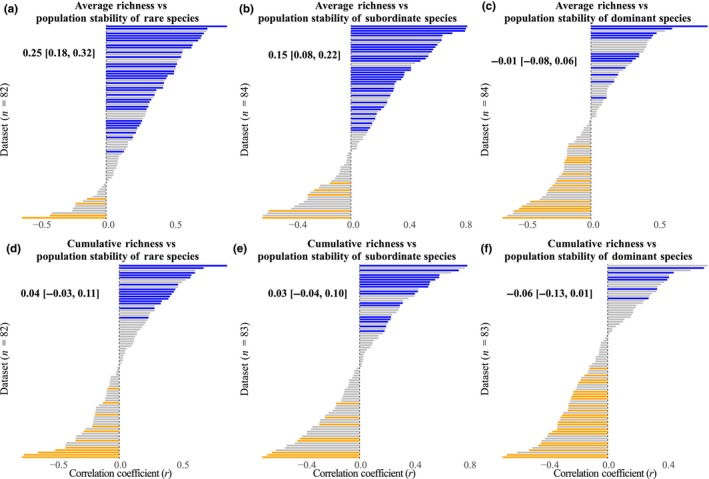
Histograms of the correlation coefficients (*r*) for each dataset between average richness and mean population stability of rare species (a), subordinate species (b), and dominant species (c), and between cumulative richness and mean population stability of rare species (d), subordinate species (e), and dominant species (f). All the variables were ln‐transformed. Different numbers of datasets were used due to the selection process. Significant positive, significant negative, and nonsignificant correlations are represented by blue, orange, and gray colors, respectively. Mean correlation coefficients ($\bar{r}$) and 95% confidence intervals from linear mixed‐effects models are also shown within each panel.

Our results revealed that the diversity–population stability relationship varied among dominant, subordinate, and rare species (Table S5). The average correlation between average richness and population stability of rare species was positive ($\bar{r}$ = 0.25, *P* < 0.001). Sixty‐seven of the 82 datasets were positive, with 31 of these being significant (Fig. 3a). Similarly, average richness was positively correlated with population stability of subordinate species ($\bar{r}$ = 0.15, *P* < 0.001; Fig. 3b). However, the number of positive and negative relationships between average richness and population stability of dominant species were similar across datasets (Fig. 3c). Using cumulative richness increased the number of negative relationships, leading to comparable amounts of positive and negative relationships for the population stability of rare (Fig. 3d) and subordinate species (Fig. 3e) but a marginally significantly negative relationship for the population stability of dominant species ($\bar{r}$ = −0.06, *P* = 0.086; Fig. 3f). The *r* values for diversity vs unweighted population stability were strongly correlated with those for diversity vs population stability of rare species (*R* ^2^ = 0.73, *P* < 0.001; Fig. S3A). By contrast, the *r* values for diversity vs weighted population stability were strongly correlated with those for diversity vs population stability of dominant species (*R* ^2^ = 0.62, *P* < 0.001; Fig. S3F).

**Fig. 4:**
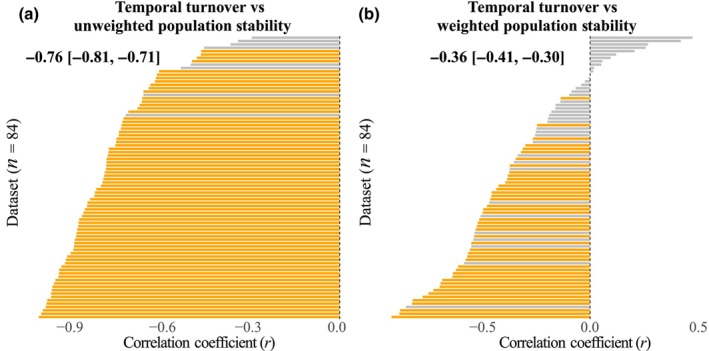
Histograms of the correlation coefficients (*r*) for each dataset between temporal turnover and unweighted population stability (a), and weighted population stability (b). All the variables were ln‐transformed. Significant positive, significant negative, and nonsignificant correlations are represented by blue, orange, and gray colors, respectively. Mean correlation coefficients ($\bar{r}$) and 95% confidence intervals from linear mixed‐effects models are also shown within each panel.

**Fig. 5:**
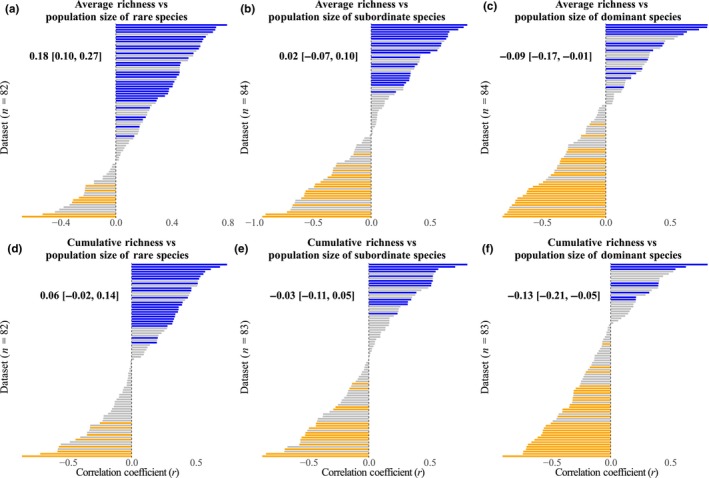
Histograms of the correlation coefficients (*r*) for each dataset between average richness and mean population size of rare species (a), subordinate species (b), and dominant species (c), and between cumulative richness and mean population size of rare species (d), subordinate species (e), and dominant species (f). All the variables were ln‐transformed. Mean population size was scaled within each data type. Different numbers of datasets were used due to the selection process. Significant positive, significant negative, and nonsignificant correlations are represented by blue, orange, and gray colors, respectively. Mean correlation coefficients ($\bar{r}$) and 95% confidence intervals from linear mixed‐effects models are also shown within each panel.

**Fig. 6:**
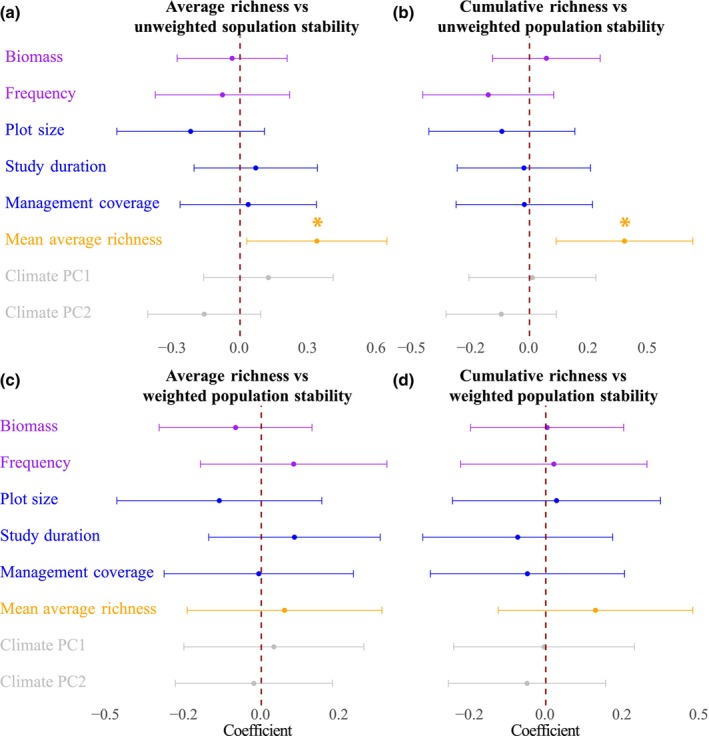
Influence of multiple abiotic and biotic drivers on the correlation coefficient (*r*) between: (a) average richness and unweighted population stability, (b) cumulative richness and unweighted population stability, (c) average richness and weighted population stability, and (d) cumulative richness and weighted population stability. Colors represent different driver categories: type of abundance metric (purple; cover as the reference level), plot attributes (blue), biotic attributes (orange), and climatic variables (gray). Estimated coefficients and their 95% confidence intervals are shown; significant effects (*P* < 0.05) are indicated by *.

Our results showed a strong negative mean correlation between temporal turnover and unweighted population stability ($\bar{r}$ = −0.76, *P* < 0.001; Fig. 4a; Table S6) but a weaker mean correlation with weighted population stability ($\bar{r}$ = −0.36, *P* < 0.001; Fig. 4b; Table S6). Analysis of mean population size against diversity showed different patterns for dominant, subordinate, and rare species (Table S7). Mean population size of rare species increased with average richness ($\bar{r}$ = 0.18, *P* < 0.001; Fig. 5a), but not with cumulative richness (Fig. 5d). For subordinate species, we found no change in population size with either average (Fig. 5b) or cumulative richness (Fig. 5e). For dominant species, however, mean population size decreased with both average ($\bar{r}$ = −0.09, *P* = 0.039; Fig. 5c) and cumulative richness ($\bar{r}$ = −0.13, *P* = 0.003; Fig. 5f). Our results also indicated that the relationships between species diversity and population stability were consistent (Fig. 6). Among the tested variables, only mean average richness significantly drove the relationship between diversity and unweighted population stability to be more positive. This positive effect indicated that the link between diversity and unweighted population stability was stronger at sites with more species.

## Discussion

Our study reconciles links between plant diversity and population stability by revealing that inconsistencies in the relationship across studies stem, at least in part, from the emerging properties of diversity and population stability metrics. We used observational data because, in biodiversity experiments, the high correlation between average and cumulative richness due to weeding can obscure these relationships. While we observed a generally positive (i.e. stabilizing) relationship between average richness and unweighted population stability (metrics commonly used in field studies), our findings revealed increasingly negative (i.e. destabilizing) relationships when richness was accumulated across years and when stability metrics accounted for species dominance. This suggests that changing species composition as a result of both deterministic and stochastic processes causes less stability in diverse plant communities.

### Average vs cumulative richness

The relationships between plant diversity and population stability differed with the use of average vs cumulative richness. Consistent with our hypotheses, cumulative richness was negatively correlated with population stability, while average richness was more often positively correlated. These findings are supported by observational studies reporting positive associations between average richness and population stability (Houlahan *et al*., 2018; Xu *et al*., 2021), as well as by numerous experimental studies showing that increased cumulative richness reduces population stability (Tilman *et al*., 2006; Hector *et al*., 2010; Roscher *et al*., 2011). Our results suggest that the differing relationships observed with average vs cumulative richness may arise from (1) the dominance of different ecological mechanisms and (2) mathematical necessity.

Cumulative richness, which reflects the broader species pool at a location over time, plays a key role in shaping species interaction dynamics. This long‐term measure provides valuable insights into how competition develops across years (Cornell & Harrison, 2014). Interspecific competition, where dominant species exclude others by monopolizing space and resources (Tilman, 1982), can increase the variance of population abundance across years, thereby reducing overall population stability. As cumulative richness is more likely to include transient and rare species that exhibit strong temporal fluctuations over time, this will result in a negative relationship between cumulative richness and population stability. Average richness, on the other hand, reflects typical species composition and abundance at a location over time. Theoretical models predict that mean population abundance decreases with diversity if total community abundance remains constant (Tilman, 1999), yet natural communities often deviate from this pattern, with community diversity and population abundances positively covarying (Kaspari *et al*., 2000; Valone & Hoffman, 2003). In line with this point, our results showed that the population size of rare species increased with average richness. This pattern may be explained by strong facilitation or niche complementarity among rare species in diverse communities.

Our results also confirmed that the differing relationships stem partially from mathematical necessity, as evidenced by the strong, negative relationship between temporal turnover and unweighted population stability. This mechanism requires rare species to be less stable, a pattern consistent with Taylor's power law (Taylor, 1961), which posits that dominant species have higher stability when the scaling factor is < 2. A recent study using the same datasets revealed a widespread scaling effect consistent with Taylor's power law, with scaling factors predominantly < 2 (Gracia *et al*., 2026), further supporting our results.

### Unweighted vs weighted population stability

Contrary to our hypotheses, we found a more positive relationship between diversity and population stability when using unweighted population stability. This result was unexpected, as dominant species are expected to be more stable than non‐dominant species, based on Taylor's power law (Taylor, 1961). However, further analyses revealed that the relationship differs among dominant and rare species, providing a compelling explanation for this unexpected pattern. Specifically, the diversity–unweighted population stability relationship was primarily explained by rare species (*r* ^2^ = 0.73; Fig. S3A), whose population stability increased with average richness, while the diversity–weighted population stability relationship was driven by dominant species (*r* ^2^ = 0.62; Fig. S3F), for which population stability negatively correlated with cumulative richness. This suggests that in richer communities, rare species become more stable, whereas dominant species tend to deflate. These differences were partly driven by variations in population size: the population size of rare species increased with average richness, whereas the population size of dominant species decreased with both average and cumulative richness.

One potential mechanism underlying these results is plant–fungal mutualism, particularly associations with arbuscular mycorrhizal fungi (AMF). AMF are known to reduce fitness differences among coexisting plant species by enhancing nutrient and water uptake, thereby promoting coexistence (Willing *et al*., 2024). Under this scenario, rare species gain better access to resources, leading to higher mean abundance and greater stability. Conversely, dominant species may experience a dilution of their competitive advantage, resulting in lower mean abundance and reduced stability. This mechanism is expected to operate more strongly in AMF‐dominated grasslands than in ectomycorrhizal (ECM)‐dominated forests. Supporting this interpretation, we found that the change in correlation coefficient from unweighted to weighted population stability for average richness was nearly twofold greater in grasslands (Estimate = −0.199) than in forests (Estimate = −0.110). However, this difference was not statistically significant (*F*_1,61_ = 0.79, *P* = 0.379), likely due to the lower number of forest datasets (*n* = 6) compared with grassland datasets (*n* = 57). Future research could further evaluate this pattern using broader and more comprehensive datasets. Consequently, the relationship between diversity and population stability became more positive when using unweighted population stability. Our study demonstrates, for the first time, that the relationship between diversity and population stability differs significantly between dominant and rare species, providing new insights into the mechanisms underlying diversity–stability relationships.

### Drivers of the diversity–population stability relationship

Previous research suggests that different measures of abundance (e.g. cover or biomass) can influence population stability (Pan *et al*., 2024), thereby influencing the diversity–population stability relationship. Cottingham *et al*. (2001) also suggested that diversity–stability relationships are mediated by factors varying across community types. By contrast, our results showed that these relationships remained consistent across abiotic and biotic gradients. We found that only mean average richness positively influenced the diversity–unweighted population stability relationship, with sites of higher richness tending to support more rare species, thereby enhancing stability. This effect was nonsignificant for weighted population stability, which was more influenced by dominant species. This finding again supports our previous results that rare species tend to have positive diversity–stability relationships.

In line with previous research (Houlahan *et al*., 2018), our results confirm that, even after accounting for different diversity and stability metrics, substantial variation remains in the strength and direction of diversity–population stability relationships. This residual variation likely reflects additional ecological influences not accounted for in our models, such as variation in soil properties and AMF. These findings underscore the need for integrative approaches that combine richness metrics with ecological context. Future research should identify the ecological and environmental conditions under which diversity–stability relationships become more consistent and predictable across ecosystems.

### Conclusions

To our knowledge, our study is the first to explore the joint effects of the emerging properties of different species diversity and population stability metrics on the diversity–population stability relationship in natural communities. We thereby demonstrate that the inconsistencies in previous findings can be partially reconciled by accounting for the influence of different metrics. These findings offer valuable insights into the longstanding diversity–stability debate and have important implications for conservation strategies.

## Competing interests

The authors declare no competing interests.

## Author contributions

XP and FB conceived the initial ideas; JL, MB, SC, JD, FE, FF, OG, DG, LG, CG, AG, LH, SH, VL, XL, FL, MM, RM, RP, AP, BP, JP, VP, MR, WS, JS, MS, EV, VV, DW, SW, BW, TY, FY ZZ, FB contributed to the LOTVS database. XP, YH and FB designed the methodology, analyzed the data with the help of JL and SW; XP, YH and FB led the writing of the manuscript. All authors contributed critically to the drafts and gave final approval for publication.

## Disclaimer

The New Phytologist Foundation remains neutral with regard to jurisdictional claims in maps and in any institutional affiliations.

## Supporting information


**Fig. S1** Map showing the geographical locations of the datasets included in LOTVS.
**Fig. S2** Change in correlation coefficient (*r*) across richness and population stability metrics.
**Fig. S3** Correlations between overall and subgroups diversity–population stability relationships.
**Notes S1** Why do we expect that species with frequent absences are typically those with lower abundance and stability?
**Table S1** Effects of diversity and population stability metrics on the correlation coefficient (*r*).
**Table S2** Effects of diversity and population stability metrics on the correlation coefficient (*r*) with the detrending method.
**Table S3** Effects of diversity and population stability metrics on the correlation coefficient (*r*) with the 10% threshold method.
**Table S4** Effects of diversity and population stability metrics on the correlation coefficient (*r*) with the 20% threshold method.
**Table S5** Mean correlation coefficient ($\bar{r}$) with combination of diversity and mean population stability of subgroups.
**Table S6** Mean correlation coefficient ($\bar{r}$) with temporal turnover and population stability.
**Table S7** Mean correlation coefficient ($\bar{r}$) with combination of diversity and mean population size of different groups.
Please note: Wiley is not responsible for the content or functionality of any Supporting Information supplied by the authors. Any queries (other than missing material) should be directed to the *New Phytologist* Central Office.

## Data Availability

The data and code supporting these results have been archived in the Zenodo repository, accessible at: doi: 10.5281/zenodo.15439499.
