# Supplementary material for: Reconciling links between diversity and population stability across global plant communities
Source: New Phytol. 2026 Jan 16;250(1):154–65. doi: 10.1111/nph.70921 (PMC12961246; doi:10.1111/nph.70921)
Supplement: Supplementary file 1 — Fig. S1 Map showing the geographical locations of the datasets included in LOTVS. Fig. S2 Change in correlation coefficient (r) across richness and population stability metrics. Fig. S3 Correlations between overall and subgroups diversity–population stability relationships. Notes S1 Why do we expect that species with frequent absences are typically those with lower abundance and stability? Table S1 Effects of diversity and population stability metrics on the correlation coefficient (r). Table S2 Effects of diversity and population stability metrics on the correlation coefficient (r) with the detrending method. Table S3 Effects of diversity and population stability metrics on the correlation coefficient (r) with the 10% threshold method. Table S4 Effects of diversity and population stability metrics on the correlation coefficient (r) with the 20% threshold method. Table S5 Mean correlation coefficient (r¯) with combination of diversity and mean population stability of subgroups. Table S6 Mean correlation coefficient (r¯) with temporal turnover and population stability. Table S7 Mean correlation coefficient (r¯) with combination of diversity and mean population size of different groups. Please note: Wiley is not responsible for the content or functionality of any Supporting Information supplied by the authors. Any queries (other than missing material) should be directed to the New Phytologist Central Office. [file NPH-250-154-s001.pdf]

New Phytologist

***New Phytologist* Supporting Information**

Article title: **Reconciling links between diversity and population stability across global plant communities**

Authors: Xiaobin Pan, Yann Hautier, Jan Lepš, Shaopeng Wang, Kathryn E. Barry, Manuele Bazzichetto, Stefano Chelli, Jiří Doležal, Nico Eisenhauer, Franz Essl, Felícia M. Fisher, Oscar Godoy, Daniel Gómez-García, Lars Götzenberger, Clara Gracia, Anaclara Guido, Lauren M. Hallett, Susan Harrison, Miao He, Andrew Hector, Pubin Hong, Forest Isbell, George A. Kowalchuk, Victor Lecegui, Xiaofei Li, Maowei Liang, Frédérique Louault, Maria Májeková, Rob Marrs, Neha Mohanbabu, Akira S Mori, Robin J. Pakeman, Alain Paquette, Begoña Peco, Josep Peñuelas, Valério D. Pillar, Marta Rueda, Wolfgang Schmidt, Jules Segrestin, Marta Gaia Sperandii, Enrique Valencia, Vigdis Vandvik, Shengnan Wang, David Ward, Susan Wiser, Ben A. Woodcock, Chong Xu, Truman Young, Fei-Hai Yu, Liting Zheng, Zhiwei Zhong, Francesco de Bello

Article acceptance date: 18 December 2025

The following Supporting Information is available for this article:

Fig. S1 Map showing the geographical locations of the data sets included in LOTVS.

Fig. S2 Change in correlation coefficient (*r*) across richness and population stability metrics.

Fig. S3 Correlations between overall and subgroups diversity-population stability relationships.

Table S1 Effects of diversity and population stability metrics on the correlation coefficient (*r*).

Table S2 Effects of diversity and population stability metrics on the correlation coefficient (*r*) with the detrending method.

Table S3 Effects of diversity and population stability metrics on the correlation coefficient (*r*) with the 10% threshold method.

Table S4 Effects of diversity and population stability metrics on the correlation coefficient (*r*) with the 20% threshold method.

Table S5 Mean correlation coefficient ($\bar{r}$) with combination of diversity and mean

population stability of subgroups.

Table S6 Mean correlation coefficient ($\bar{r}$) with temporal turnover and population stability.

Table S7 Mean correlation coefficient ($\bar{r}$) with combination of diversity and mean population size of different groups.

Note S1 Why do we expect that species with frequent absences are typically those with lower abundance and stability?

Figure S1. Map showing the geographical locations of the data sets included in LOTVS.

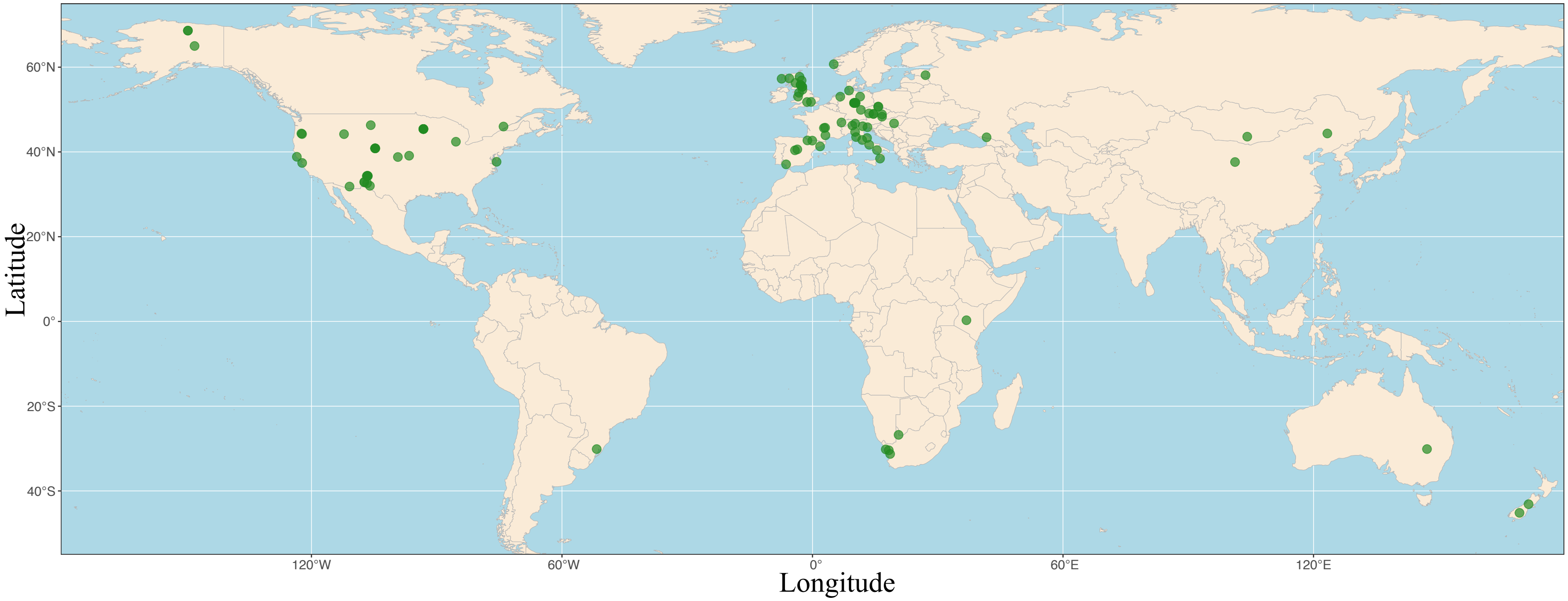

Figure S2. Change in correlation coefficient (r) from average to cumulative richness for unweighted population stability (A) and weighted population stability (B), and from unweighted to weighted population stability for average richness (C) and cumulative richness (D). Positive and negative changes are represented by blue and orange, respectively.

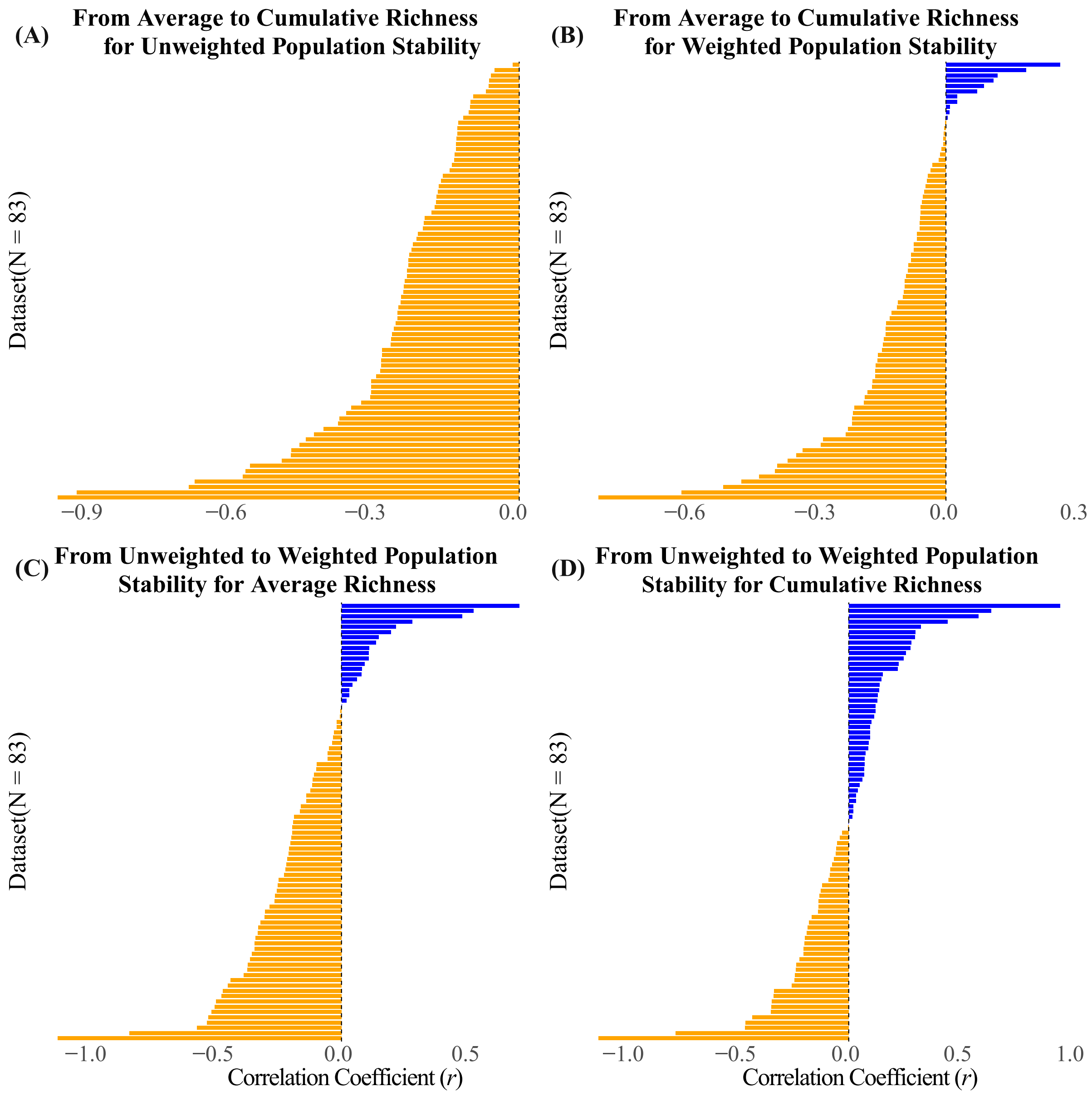

New Phytologist

Figure S3. Relationships between the *r* values for diversity vs. unweighted population stability and the *r* values for diversity vs. mean population stability of rare species (A), subordinate species (B), and dominant species (C), and between the *r* values for diversity vs. weighted population stability and the *r* values for diversity vs. mean population stability of rare species (D), subordinate species (E) and dominant species (F).

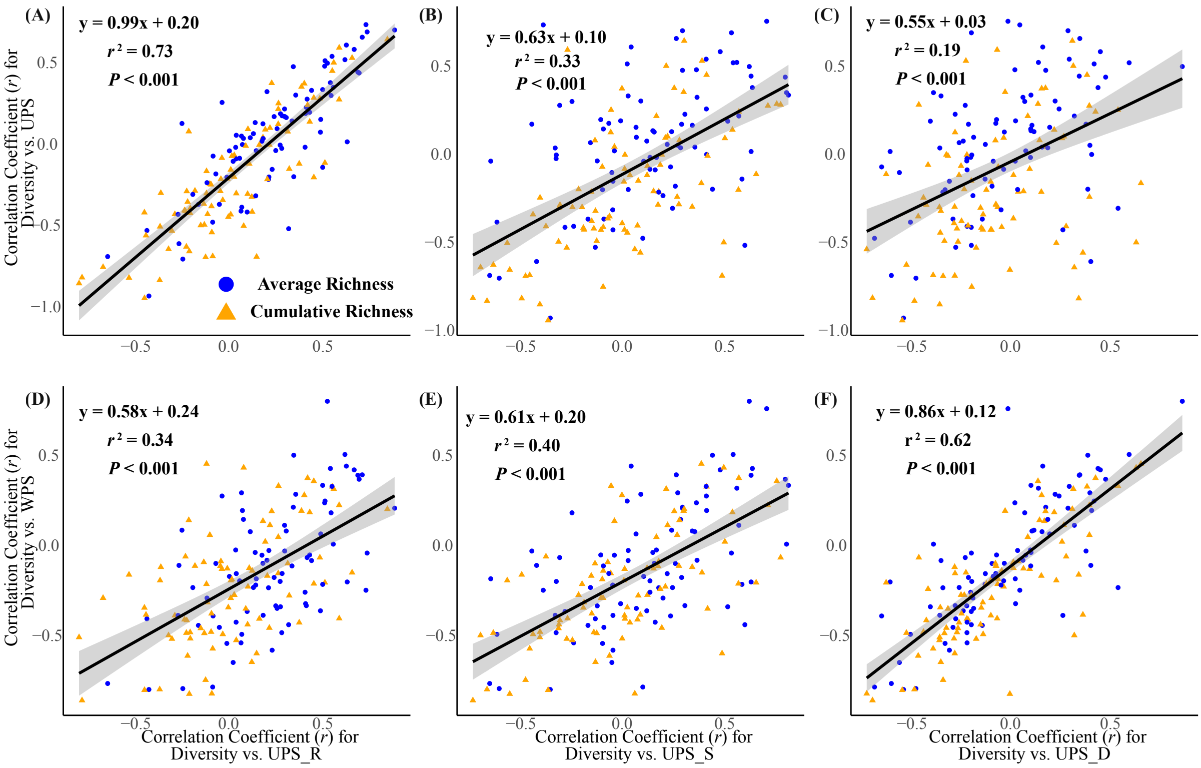

Table S1. Results (A) of linear mixed-effect models testing the effects of diversity (average and cumulative richness) and population stability metrics (unweighted mean and weighted mean) on the correlation coefficient (*r*), with dataset as a random factor. Evaluation of mean correlation coefficient ($\bar{r}$) with combination of diversity and population stability metrics based on linear mixed-effect models (B) and random-effects meta-regression models (C). UPS: unweighted population stability; WPS: weighted population stability.

**(A)** Response variable	*F* value	DF	*P*-value
Diversity Metric	101.42	1, 246.80	< **0.001**
Population Stability Metric	27.54	1, 246.41	< **0.001**
Interaction	12.73	1, 246.41	< **0.001**

**(B)** Diversity Metric	Population Stability Metric	$\bar{r}$	*P*-value
Average richness	UPS	0.086	**0.026**
Cumulative richness	UPS	-0.178	< **0.001**
Average richness	WPS	-0.085	**0.030**
Cumulative richness	WPS	-0.210	< **0.001**

**(C)** Diversity Metric	Population Stability Metric	Lower. CL	$\bar{r}$	Upper. CL
Average richness	UPS	-0.014	0.078	0.169
Cumulative richness	UPS	-0.304	-0.214	-0.121
Average richness	WPS	-0.197	-0.112	-0.026
Cumulative richness	WPS	-0.330	-0.255	-0.176

Table S2. Results (A) of linear mixed-effect models testing the effects of diversity (average and cumulative richness) and population stability metrics (unweighted mean and weighted mean) on the correlation coefficient (*r*), with dataset as a random factor. Evaluation of mean correlation coefficient ($\bar{r}$) with combination of diversity and population stability metrics based on linear mixed-effect models (B) and random-effects meta-regression models (C). UPS: unweighted population stability; WPS: weighted population stability. Population stability was detrended using the three-term local variance method.

**(A)**	Response variable	*F* value	DF	*P*-value
	Diversity Metric	43.83	1, 246.8	**< 0.001**
	Population Stability Metric	9.86	1, 246.1	**0.002**
	Interaction	7.73	1, 246.1	**0.005**
**(B)**	Diversity Metric	Population Stability Metric	$\bar{r}$	*P*-value
	Average richness	UPS	0.036	0.299
	Cumulative richness	UPS	-0.172	**< 0.001**
	Average richness	WPS	-0.095	**0.007**
	Cumulative richness	WPS	-0.180	**< 0.001**

Table S3. Results (A) of linear mixed-effect models testing the effects of diversity (average and cumulative richness) and population stability metrics (unweighted mean and weighted mean) on the correlation coefficient (*r*), with dataset as a random factor. Evaluation of mean correlation coefficient ($\bar{r}$) with combination of diversity and population stability metrics based on linear mixed-effect models (B) and random-effects meta-regression models (C). UPS: unweighted population stability; WPS: weighted population stability. Species were filtered with 10% threshold.

**(A)**	Response variable	*F* value	DF	*P*-value
	Diversity Metric	122.00	1, 249	< **0.001**
	Population Stability Metric	31.90	1, 249	< **0.001**
	Interaction	33.19	1, 249	< **0.001**
**(B)**	Diversity Metric	Population Stability Metric	$\bar{r}$	*P*-value
	Average richness	UPS	0.213	< **0.001**
	Cumulative richness	UPS	-0.261	< **0.001**
	Average richness	WPS	-0.109	**0.006**
	Cumulative richness	WPS	-0.258	< **0.001**

Table S4. Results (A) of linear mixed-effect models testing the effects of diversity (average and cumulative richness) and population stability metrics (unweighted mean and weighted mean) on the correlation coefficient (r), with dataset as a random factor. Evaluation of mean correlation coefficient ($\bar{r}$) with combination of diversity and population stability metrics based on linear mixed-effect models (B) and random-effects meta-regression models (C). UPS: unweighted population stability; WPS: weighted population stability. Species were filtered with 20% threshold.

**(A)**	Response variable	*F* value	DF	*P*-value
	Diversity Metric	129.50	1, 249	< **0.001**
	Population Stability Metric	28.57	1, 249	< **0.001**
	Interaction	22.95	1, 249	< **0.001**
**(B)**	Diversity Metric	Population Stability Metric	$\bar{r}$	*P*-value
	Average richness	UPS	0.114	**0.003**
	Cumulative richness	UPS	-0.231	< **0.001**
	Average richness	WPS	-0.102	**0.007**
	Cumulative richness	WPS	-0.243	< **0.001**

Table S5. Evaluation of mean correlation coefficient ($\bar{r}$) with combination of diversity (average and cumulative richness) and mean population stability of different groups (rare, subordinate, and dominant species) based on linear mixed-effect models (A) and random-effects meta-regression models (B).

**(A)** Diversity Index	Mean Population Stability	$\bar{r}$	*P*-value
Average richness	Rare species	0.253	**< 0.001**
Average richness	Subordinate species	0.145	**< 0.001**
Annual richness	Dominant species	-0.005	0.882
Cumulative richness	Rare species	0.043	0.235
Cumulative richness	Subordinate species	0.028	0.430
Cumulative richness	Dominant species	-0.062	0.086

**(B)** Diversity Index	Mean Population Stability	Lower. CL	$\bar{r}$	Upper. CL
Average richness	Rare species	0.190	0.266	0.338
Average richness	Subordinate species	0.093	0.174	0.253
Annual richness	Dominant species	-0.087	-0.019	0.050
Cumulative richness	Rare species	-0.023	0.057	0.136
Cumulative richness	Subordinate species	-0.044	0.037	0.118
Cumulative richness	Dominant species	-0.153	-0.088	-0.023

Table S6. Evaluation of mean correlation coefficient ($\bar{r}$) with temporal turnover (cumulative/average richness) and population stability (unweighted mean and weighted mean) based on linear mixed-effect models (A) and random-effects meta-regression models (B). UPS: unweighted population stability; WPS: weighted population stability.

**(A)** Diversity	Population stability	$\bar{r}$	*P*-value
Temporal turnover	UPS	-0.760	**< 0.001**
	WPS	-0.355	**< 0.001**

**(B)** Diversity	Population Stability	Lower. CL	$\bar{r}$	Upper. CL
Temporal turnover	UPS	-0.828	-0.800	-0.769
	WPS	-0.484	-0.422	-0.355

Table S7. Evaluation of mean correlation coefficient ($\bar{r}$) with combination of diversity (average and cumulative richness) and mean population size of different groups (rare, subordinate, and dominant species) based on linear mixed-effect models (A) and random-effects meta-regression models (B).

**(A)** Diversity Index	Mean Population Size	$\bar{r}$	*P*-value
Average richness	Rare species	0.184	**< 0.001**
Average richness	Subordinate species	0.015	0.719
Average richness	Dominant species	-0.087	**0.039**
Cumulative richness	Rare species	-0.029	0.491
Cumulative richness	Subordinate species	0.062	0.141
Cumulative richness	Dominant species	-0.128	**0.003**

**(B)** Diversity Index	Mean Population Size	Lower. CL	$\bar{r}$	Upper. CL
Average richness	Rare species	0.131	0.210	0.287
Average richness	Subordinate species	-0.072	0.033	0.137
Annual richness	Dominant species	-0.212	-0.114	-0.014
Cumulative richness	Rare species	0.017	0.099	0.180
Cumulative richness	Subordinate species	-0.102	-0.012	0.079
Cumulative richness	Dominant species	-0.240	-0.154	-0.066

Note S1. Why do we expect that species with frequent absences are typically those with lower abundance and stability?

If the abundances in years when a species is present remain constant (an oversimplification, but plausible in cases where a species is either absent or recorded at the minimum detectable level, typically 0.1% cover for low-abundance species), then the coefficient of variation (CV) can be expressed as

$$CV = \sqrt{\frac{n}{n-1}} \times \sqrt{\frac{q}{1-q}}$$

where q represents the proportion of absences in the time series, and n denotes the length of the time series.

Based on the formula, we expect species to be less stable when there are more absences across time series.
